# Clinical implications of determination of safe surgical margins by using a combination of CT and 18FDG-positron emission tomography in soft tissue sarcoma

**DOI:** 10.1186/1471-2474-12-166

**Published:** 2011-07-21

**Authors:** Masahiro Yokouchi, Mikio Terahara, Satoshi Nagano, Yoshiya Arishima, Michihisa Zemmyo, Takako Yoshioka, Akihide Tanimoto, Setsuro Komiya

**Affiliations:** 1 Department of Orthopaedic Surgery, Graduate School of Medical and Dental Sciences, Kagoshima University, Kagoshima, Japan; 2 Molecular and Cellular Pathology, Graduate School of Medical and Dental Sciences, Kagoshima University, Kagoshima, Japan

## Abstract

**Background:**

To determine safe surgical margins for soft tissue sarcoma, it is essential to perform a general evaluation of the extent of tumor, responses to auxiliary therapy, and other factors preoperatively using multiple types of diagnostic imaging. 18F-fluorodeoxyglucose positron emission tomography/computed tomography (FDG-PET/CT) is a tool for diagnostic imaging that has recently spread rapidly in clinical use. At present, the roles played by FDG-PET/CT in determination of margins for surgical resection of sarcoma are unclear. The present study was undertaken to explore the roles of FDG-PET/CT in determination of surgical margins for soft tissue sarcoma and to examine whether PET can serve as a standard means for setting the margins of surgical resection during reduced surgery.

**Methods:**

The study involved 7 patients with sarcoma who underwent surgery in our department and in whom evaluation with FDG-PET/CT was possible. Sarcoma was histologically rated as MFH in 6 cases and leiomyosarcoma in 1 case. In all cases, sarcoma was superficial (T1a or T2a). The tumor border was defined by contrast-enhanced MRI, and SUVs were measured at intervals of 1 cm over a 5-cm long area from the tumor border. Mapping of viable tumor cells was carried out on whole-mount sections of resected tissue, and SUVs were compared with histopathological findings.

**Results:**

Preoperative maximum SUVs (SUV-max) of the tumor averaged 11.7 (range: 3.8-22.1). Mean SUV-max was 2.2 (range: 0.3-3.8) at 1 cm from the tumor border, 1.1 (0.85-1.47) at 2 cm, 0.83 (0.65-1.15) at 3 cm, 0.7 (0.42-0.95) at 4 cm, and 0.64 (0.45-0.82) at 5 cm. When resected tissue was mapped, tumor cells were absent in the areas where SUV-max was below 1.0.

**Conclusions:**

Our findings suggest that a safe surgical margin free of viable tumor cells can be ensured if the SUV cut-off level is set at 1.0. FDG-PET/CT is promising as a diagnostic imaging technique for setting of safe minimal margins for surgical resection of soft tissue sarcoma.

## Background

In surgical treatment of soft tissue sarcoma, it is as a rule necessary to remove the tumor in a reliable fashion with surgical margins at which recurrence is unlikely. With this principle, Enneking et al. [1] proposed extensive resection based on the "compartment" concept. Later, Kawaguchi et al. [2] proposed curative extensive resection, adopting the concept of "barrier on the basis of tumor-resistant tissue." Guidelines for treatment of varying stages of soft tissue sarcoma such as the National Comprehensive Cancer Network (NCCN) Guidelines are available, and techniques for soft tissue sarcoma resection have been relatively well established. In the past, contrast-enhanced MRI was often used as a primary technique for determining surgical margins for soft tissue sarcoma. It is essential to perform general evaluation of the extent of tumor, responses to auxiliary therapy, and other factors preoperatively using multiple diagnostic imaging techniques. In the present study, we paid close attention to FDG-PET/CT, a tool for diagnostic imaging the use of which has spread rapidly in recent years. FDG-PET/CT has been shown to be useful in grading soft tissue sarcoma and evaluating responses to chemotherapy [3]. However, no report has been published concerning the effectiveness of FDG-PET/CT in determining surgical margins for soft tissue sarcoma. In the field of radiation therapy, the use of FDG-PET in combination with CT has been shown to enable efficient planning of treatment, since FDG-PET enables more accurate calculation of the actual extent of tumor than macroscopic evaluation of target tumor volume with CT or MRI [4,5]. This technique has been used effectively in clinical practice. The present study was undertaken to establish a new way of setting of surgical margins in the direction free of biological barrier tissue [2,6,7] by measurement of SUV at the tumor periphery.

## Methods

**Figure 1:**
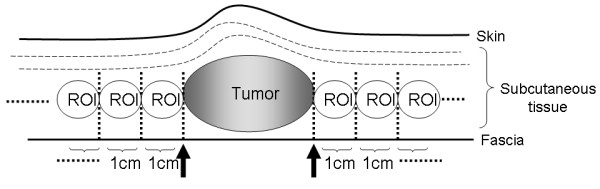
**The Setting of Regions of interest (ROIs) from the tumor border**. After the tumor border was defined by contrast-enhanced MRI, regions of interest (ROIs) were set at intervals of 1 cm for the 5 cm long region from the tumor border in a direction free of biological barrier. SUV data by preoperative FDG-PET/CT in these regions were analyzed using VISIO KEOPSYS VIEWER (CODONICS: USA). Measurement was performed in at least two directions for each case. (Black arrow: tumor border defined by contrast-enhanced MRI).

**Figure 2:**
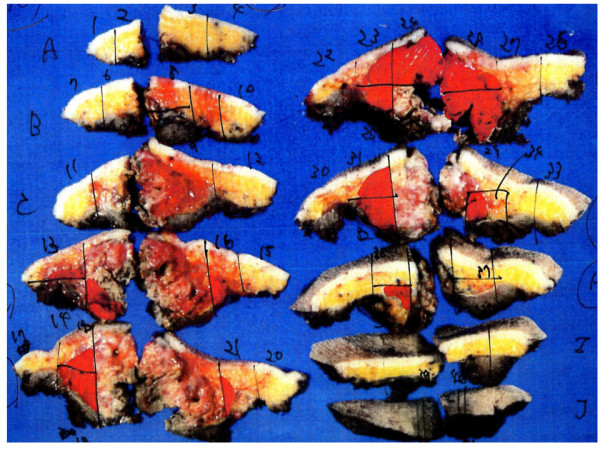
**Mapping of viable tumor cells on the whole cut section**. Extensive resection of tumor was carried out with a margin of 3-5 cm from the tumor periphery. Mapping of viable tumor cells was carried out on whole sections of resected tissue (viable cells mapped in red color).

The study involved 7 patients with subcutaneous soft tissue sarcoma who underwent treatment in our department between 2008 and 2009 and in whom evaluation by FDG-PET/CT was possible. They included 3 males and 4 females with a mean age of 70.4 years (range: 60-79). The tumor was histologically rated as MFH in 6 cases and leiomyosarcoma in 1 case (Table 1). For these cases, we determined tumor borders by using conventional contrast-enhanced MRI, and resection was planned to secure surgical margins between 3 and 5 cm from tumor borders determined by contrast-enhanced MRI. We selected 2 directions without biological barriers from among sagittal, axial, and coronal directions in tumor borders determined by preoperative contrast-enhanced MRI, and we measured SUV values of soft tissues that were indicated to be outside the tumor borders by contrast-enhanced MRI. SUV data by preoperative FDG-PET/CT were analyzed using VISIO KEOPSYS VIEWER (CODONICS: USA). In other words, we determined that the regions of interest (ROI) were at a distance of 5 cm and 1-cm interval from the tumor borders by contrast-enhanced MRI, and measured their SUV-max value (Figure 1). After extensive resection of the tumors, we performed mapping of neoplastic cells using whole area histological specimens of the resected tissues and compared with the preoperative SUV values (Figure 2).

**Table 1:** Patient and tumour details

Sex/Age	Histologic type	Location	SUV-max	Follow-up period	Recurrence	Prognosis
Male/70	MFH	Thigh	3.9	2y5M	(-)	DF
Male/73	MFH	Chest	12.9	1y1M	(-)	AWD
Male/60	MFH	Shoulder	10.5	1y1M	(-)	DF
Female/64	MFH	Thigh	3.8	2y3M	(-)	DF
Female/77	MFH	Forearm	13.8	2y2M	(-)	DF
Female/79	MFH	Buttock	14.9	1y	(-)	DF
Female/70	Leiomyo Sarcoma	Thigh	22.1	2y1M	(-)	DF

DF: disease free, AWD: alive with disease

## Results

Preoperative SUV-max of the tumor averaged 11.7 (range: 3.8-22.1). Mean SUV-max was 2.2 (range: 0.3-3.8) at 1 cm from the tumor border, 1.1 (0.85-1.47) at 2 cm, 0.83 (0.65-1.15) at 3 cm, 0.7 (0.42-0.95) at 4 cm, and 0.64 (0.45-0.82) at 5 cm (Table 2). When the resected tissue was mapped, the regions not more than 1.0 in SUV-max were free of tumor cells (tumor positive rate: 0%). The tumor positive rate was 25% for regions with SUV-max between 1.0 and 2.0 and 80% for those with SUV-max not less than 2.0 (Table 3). At present, 1Y to 2Y5M after the start of postoperative follow-up, all patients are alive without recurrence (Table 1).

**Table 2:** Preoperative SUV values

Distance from the tumor border	SUV-max (range)
Tumor	11.7 (3.8 ~ 22.1)
0 ~ 1 cm	2.2 (0.3 ~ 3.8)
1 ~ 2 cm	1.1 (0.85 ~ 1.47)
2 ~ 3 cm	0.83 (0.65 ~ 1.15)
3 ~ 4 cm	0.7 (0.42 ~ 0.95)
4 ~ 5 cm	0.64 (0.45 ~ 0.82)

**Table 3:** The rates of viable tumor cells

SUV-max	Tumor positive rate
< 1.0	0%
1.0 ~ 2.0	25%
> 2.0	80%

## Discussion

In 1980, Enneking et al. [1] proposed a technique of extensive resection by which dissection along the longitudinal direction of muscle is performed from origin to end and dissection along the transverse plane is performed in a compartment-wise fashion. Their technique, however, featured problems such as excessively wide resection and postoperative loss of function. In Japan, Kawaguchi et al. [2] proposed in 1982 a technique for curative extensive resection, adopting the concept of barrier based on tumor resistance of fascia, periosteum, cartilage tissue, and other tissues. Subsequently, data on surgical margins for a very large number of cases were analyzed to determine optimal methods of setting the surgical margins depending on tumor type, degree of histological malignancy, growth profile (invasive/noninvasive), recurrence, need for additional surgery, presence/absence of preoperative therapy, and other factors. On the basis of these analyses, extensive resection was defined as resection of the 1-4 cm area from the tumor-reactive layer and curative extensive resection as resection for 5 cm or more [6,7]. The guidelines published in 2010 by the National Comprehensive Cancer Network (NCCN) proposed methods of treatment for varying stages of disease, and indicated that in case of Stage 1 tumor resection with a margin not less than 1 cm, the local rate of recurrence is low and close follow-up without active treatment suffices after resection while in case with an eventual margin less than 1 cm, addition of auxiliary radiotherapy is examined. In cases of Stage II/III tumor resection should as a rule be extensive (ensuring a margin of several centimeters free of tumor cells in all directions) and combined with preoperative/postoperative adjuvant chemotherapy and radiotherapy.

As indicated above, extensive resection with a safe margin has been established. Furthermore, it has been reported that adjuvant chemotherapy significantly extends the length of time until local recurrence or distant metastasis of non-small round cell soft tissue sarcoma and significantly improves the 10-year disease-free survival rate [8]. Furthermore, outcome of treatment was improved by radiation, which contributed to preservation of affected limb function through tumor size reduction when performed preoperatively and through improvement of local control rate when performed postoperatively [9,10]. However, large tumors, those involving the nerves/blood vessels, tumors resistant to auxiliary therapy, and some other types of tumors still require sufficiently extensive resection, including normal tissue as well, accompanied by reconstruction-assisting surgery using flaps, vascular prostheses, and other techniques. These methods of treatment involve problems such as high cost, long time of surgery, and long hospital stay [11,12]. It is therefore desirable to explore the possibility of reducing surgical margins while observing the principle of ensuring radical treatment of tumor. Under these circumstances, we have recently begun to pay close attention to FDG-PET/CT, which has recently been reported to be useful in grading soft tissue sarcoma and evaluating responses to chemotherapy [3]. In the field of radiotherapy, the effectiveness of FDG-PET as a means of determining the area of irradiation for various types of cancer has been increasingly evaluated. When radiotherapy is planned, the area for irradiation, i.e., the target volume, is first decided. The target volume can be divided into gross tumor volume (GTV), clinical target volume (CTV), internal target volume (ITV), and planning target volume (PTV). When the target volume is determined, assessment of lesion extent is most important, making use not only of visual inspection and palpation but also CT, MRI, FDG-PET, gastrointestinal radiography, and other imaging methods. Some investigators reported that more efficient treatment planning is possible with the use of FDG-PET in combination with CT, since FDG-PET enables more accurate calculation of the actual extent of tumor compared to GTV based on CT or MRI [4], while other investigators have reported that GTV could be significantly reduced with the use of FDG-PET compared to the use of CT scans [13], thus demonstrating the usefulness of FDG-PET. There is a report that because glucose consumption by tumor stroma may be reduced by aerobic metabolism, determination of the outline of the target cancer solely on the basis of FDG during radiotherapy planning may underestimate real tumor volume [14], indicating the need for adequate evaluation of the validity of FDG in treatment planning.

Although FDG-PET/CT is a powerful means of diagnostic imaging the use of which has spread rapidly in recent years, its precise roles in setting of margins for surgical resection of sarcoma have yet to be clearly determined. In the present study, areas with SUV-max less than 1.0 were free of viable tumor cells. This finding suggests that safe surgical margins, ensuring the absence of viable tumor cells, may be selected if the cut-off SUV-max level is set at 1.0. Although the present study was confined to cases of subcutaneous soft tissue sarcoma, we plan to study the applicability of this technique to evaluation of sarcoma around major nerves, evaluation of bone invasion of sarcoma, and other types of tissue. A limitation of this study is that the number of cases is as small as 7. This is because the incidence of sarcoma is overwhelmingly low compared with that of cancer; therefore, it was difficult to design a large-scale study since our study was limited to subcutaneous sarcomas. In the present study, SUV-max can vary greatly among tumors of the same type. It is desirable to examine a larger number of cases to determine whether the cut-off SUV-max level can be uniformly set at 1.0 in all cases including those with tumor the center of which has a low SUV-max. Hence, at the present stage, our study results may be presented as a case report. In the future, we need to collect a variety of cases from multi-institutions as well as a single institution and further accumulate reliable data. Furthermore, in cases in which the body position during FDG-PET/CT differs from that during operation, the surgeon needs to take into account possible discrepancies between SUV data and actual tumor location. In recent years, FLT-PET using fluorothymidine (FLT) as a tracer has been performed to differentiate between inflammatory and tumor lesions. Thymidine is one of the elements present in the DNA, and its incorporation into cells is known as an indicator for cellular proliferation. FLT is an analog of thymidine. FLT is phosphorylated by a thymidine salvage pathway; however, it is not incorporated into nuclei. Malignant tumors show rapid cell proliferation and enhanced nucleic acid synthesis. Because the accumulation rate of FLT in inflammatory sites is considered to be lower than that of FDG, FLT might be more useful for determining the surgical margins of tumors.

## Conclusions

Our findings suggest that a safe surgical margin free of viable tumor cells can be ensured if the SUV cut-off level is set at 1.0. FDG-PET/CT is promising as a diagnostic imaging technique for setting of safe minimal margins for surgical resection of soft tissue sarcoma.

## Competing interests

The authors declare that they have no competing interests.

## Authors' contributions

MT, SN, TY and AT participated in the design of the study and performed the histological analysis. MY and YA participated in the treatment of the case, including the surgery and drafted the manuscript. MZ and SK conceived of the study, and participated in its design and coordination and helped to draft the manuscript.

All authors read and approved the final manuscript.

## Pre-publication history

The pre-publication history for this paper can be accessed here:

http://www.biomedcentral.com/1471-2474/12/166/prepub
